# Novel bioactive extract from yarrow obtained by the supercritical antisolvent-assisted technique inhibits lipid metabolism in colorectal cancer

**DOI:** 10.3389/fbioe.2024.1256190

**Published:** 2024-03-21

**Authors:** Marta Gómez de Cedrón, Maria de las Nieves Siles-Sanchez, Diego Martín-Hernandez, Laura Jaime, Susana Santoyo, Ana Ramírez de Molina

**Affiliations:** ^1^ Molecular Oncology Group, IMDEA Food Institute, CEI UAM + CSIC, Madrid, Spain; ^2^ Institute of Food Science Research (CIAL), Universidad Autónoma de Madrid (CEI UAM + CSIC), Madrid, Spain

**Keywords:** supercritical antisolvent-assisted technique, lipid metabolism, colorectal cancer, cell bioenergetics, bioactive extracts, yarrow (*Achillea millefolium*)

## Abstract

**Background:** Altered lipid metabolism in cancer is associated to dissemination and prognosis. Bioactive compounds naturally occurring in *Achillea millefolium* L. (yarrow) have been reported to exert antitumour activities. Food biotechnology may provide on-demand mixtures of bioactive compounds with complementary activities in cancer treatment.

**Methods:** Supercritical-antisolvent-precipitation (SAS) has been applied to fractionate the bioactive compounds from an Ultrasound-Assisted-Extraction yarrow extract resulting in two extracts with distinct polarity, yarrow-precipitate-(PP) and yarrow-separator-(Sep). Total phenolic content and relevant essential oils have been characterized. Antioxidant, anti-inflammatory and antiproliferative activities have been compared. Moreover, the effect on the inhibition of colorectal cancer cells’ bioenergetics has been evaluated.

**Results:** Yarrow-PP exerted the highest antioxidant activity, even higher than the complete UAE-yarrow extract, meanwhile yarrow-Sep showed the highest anti-inflammatory activity, even higher than the complete UAE-yarrow extract. Interestingly, yarrow-Sep inhibited key lipid metabolic targets in CRC cells extensively shown to be implicated in cancer dissemination and prognosis —*SREBF1*, *FASN*, *ABCA1* and *HMGCR*— and epithelial to mesenchymal targets—*CDH1*, *ATP1B1*, *CDH2* and *Vimentin*—augmenting cell adhesion.

**Conclusions:** In summary, SAS technology has been applied to provide a novel combination of bioactive compounds, yarrow-Sep, which merits further research to be proposed as a potential complementary nutraceutical in the treatment of CRC.

## 1 Introduction

Colorectal cancer (CRC) is the third most common cancer and the second leading cause of cancer-related deaths worldwide. Metastasis is the last step of cancer, being the main responsible factor for morbidity and mortality. In fact, more than 90% of mortality associated with CRC is due to metastasis. Currently, the altered lipid metabolism in cancer is a topic of interest due to the prominent role of lipids regulating the progression in various types of tumors. Lipids and lipid-derived molecules have been shown to activate growth regulatory pathways and promote malignancy in several cancers (Aguirre-Portoles et al., 2017; Bouzas et al., 2022). During tumor development, lipid-associated alterations include i) an increase in the *de novo* lipogenesis driven by the sterol regulatory element-binding factor (*SREBF1*) and downstream molecular targets, fatty acid synthase (*FASN*), and stearoyl-CoA desaturase (*SCD-1*); ii) an increase in cholesterogenesis by means of the expression of hydroxy-methyl-glutaryl-CoA reductase (*HMGCR*), affecting plasmatic membrane fluidity and remodeling the extracellular tumor microenvironment (Aguirre-Portoles et al., 2018); and iii) an increased uptake of extracellular fatty acids by means of the increased expression of the lipoprotein low-density receptor (LDLR) and cluster differentiation 36 (CD36), which have also been demonstrated to dictate metastatic tropism in several cancers (Montero-Calle et al., 2022). Importantly, many authors have described the link between the altered expression of lipid metabolic genes and development and prognosis in cancer (Sanchez-Martinez et al., 2015; Vargas et al., 2015).

Plant-derived bioactive compounds have been extensively studied for their health-promoting benefits against chronic metabolic diseases, including obesity, type 2 diabetes mellitus (T2DM), cardiovascular diseases, dyslipidemias, and even cancer (Poorbagher et al., 2022; Tabatabaeain et al., 2022; Firouzabadi et al., 2023). All these diseases are characterized by a low grade of chronic inflammation—metainflammation—associated with systemic metabolic stress and immune system dysfunction. Importantly, natural bioactive extracts may provide potent mixtures of bioactive compounds to act synergistically by targeting many altered metabolic pathways (Gomez de Cedron et al., 2019a; Mouhid et al., 2019a; Mouhid et al., 2019b; Reguero et al., 2021a; Bouzas Munoz et al., 2021; Reguero et al., 2021b; Reguero et al., 2022). Thus, bioactive compounds naturally occurring in *Achillea millefolium* L. (yarrow), such as phenolic compounds (mainly caffeoylquinic and dicaffeoylquinic acids), and those found in the volatile oil fraction have been reported to present antitumor activities (Mohammadhosseini et al., 2017). A hydroethanolic extract of yarrow—containing 3,5-dicaffeoylquinic acid, 5-caffeoylquinic acid, luteolin-*O*-acetylhexoside, and apigenin-*O*-acetylhexoside as the main phenolic compounds—was reported to inhibit the proliferation of human colorectal adenocarcinoma cells (Pereira et al., 2018). In addition, Gupta et al. (2022) also reported chlorogenic acid (5-caffeoylquinic acid) as an effective antitumor compound (Gupta et al., 2022). Meanwhile, Garcia-Risco et al. (2017) demonstrated the antitumoral properties of a supercritical CO_2_ (SCCO_2_) extract from yarrow (SFE yarrow), rich in essential oil components, in a preclinical model of pancreatic cancer xenografts (Garcia-Risco et al., 2017; Mouhid et al., 2019a). Importantly, essential oil components in SFE yarrow inhibited the lipid metabolism in cancer cells, resulting in apoptosis. In addition, SFE yarrow improved insulin sensitivity, reduced hypercholesterolemia and liver steatosis, and increased the adipose tissue buffering capacity of circulating glucose in a preclinical model of high-fat diet (HFD)-induced obesity (Mouhid et al., 2019b).

The development of innovative approaches, such as fractionation, precipitation, and nanoencapsulation, may contribute to provide on-demand mixtures of bioactive compounds with complementary and/or synergistic activities (Oskoueian et al., 2020; Shafaei et al., 2020; Zarei et al., 2021). In this regard, supercritical antisolvent (SAS) precipitation has been demonstrated to act as a very powerful technique for the fractionation of plant extracts, providing further combinations of bioactive compounds (Mur et al., 2021). In this process, a polar liquid solution of a plant extract is continuously sprayed in a co-current with supercritical CO_2_ (SCCO_2_). Thus, more polar compounds precipitate due to their insolubility in SCCO_2_; meanwhile, less-polar compounds remain dissolved in SCCO_2_, being recovered downstream by pressure reduction of SCCO_2_ (Langa et al., 2019). At the end of the process, two fractions, the precipitate fraction and the separator fraction, are obtained containing compounds of different polarities. This technique can be considered as environmentally sustainable since it does not generate any type of waste (Gimenez-Rota et al., 2019). Moreover, CO_2_ creates an inert environment, reducing the degradation of bioactive compounds due to its low critical temperature. Thus, Villalva et al. (2019) used SAS to fractionate the bioactive compounds present in an ethanolic extract of yarrow, obtaining two fractions, one enriched in phenolic compounds and the other enriched in essential oil components (Villalva et al., 2019).

The objective of this work was to apply the SAS technology to fractionate the bioactive compounds of an ultrasound-assisted extraction from yarrow (UAE yarrow), to obtain fractions with potential activity as co-adjuvants in CRC treatment. The chemical composition of fractions obtained was characterized in terms of total phenolic compounds and essential oil composition. The antioxidant, anti-inflammatory, and antiproliferative activities were also determined. In addition, the effect on cell bioenergetics of colorectal cancer cells was evaluated.

## 2 Materials and methods

### 2.1 UAE yarrow extract

Yarrow inflorescences and upper dried leaves (batch number 395.CC.26101) were supplied by a Spanish company specialized in plant material supplies (Plantafarm, Leon, Spain). Samples were ground using a knife mill (Grindomix GM 200, Retsch, Llanera, Spain), and the particle size was reduced to <500 µm.

UAE was carried out following the method described by Villalva et al. (2021). In brief, a Branson 250 digital device (Branson Ultrasonic, Danbury, United States) with an electric power of 200 W and frequency of 60 Hz was used. Ground plant material (20 g) was extracted with ethanol (ratio of 1:10, plant:solvent) for 20 min and 60% amplitude, using a probe of ½’ diameter. Next, the samples were filtered, and ethanol was partially removed under rotary evaporation at 35°C (IKA RV 10, Madrid, Spain) to achieve a concentrated solution at 17.8 mg/mL.

### 2.2 SAS precipitation

The supercritical antisolvent precipitation was performed using the supercritical technology equipment Thar SF 2000 (Thar Technology, Pittsburgh, PA, United States) at 15 MPa and 40°C. A comprehensive explanation about the equipment and the process design used can be found elsewhere (Quintana et al., 2019).

Concisely, the experiment started by pumping SCCO_2_ (at 60 g/min flow rate along the experiment) until the pressure and temperature conditions were attained into the precipitation vessel. Then, the UAE yarrow solution (17.8 mg/mL) was pumped into this vessel at 1 mL/min for 45 min while maintaining the SCCO_2_ flow rate. After mixing, yarrow extract components not soluble in the SCCO_2_ + ethanol supercritical phase were obtained in the precipitation vessel and were collected (precipitate fraction, yarrow PP). Meanwhile, the fraction soluble in the SCCO_2_ + ethanol matrix went into the separator vessel, where ethanol and this fraction of phytochemicals were recovered. Next, additional SCCO_2_ was pumped during 15 min to remove the residual solvent from the precipitation vessel.

The samples obtained in the separator were rotary evaporated (RV 10 control VWR, IKA, Staufen, Germany) until an oleoresin product was obtained (separator fraction, yarrow Sep). Dry SAS fractions, PP, and Sep were kept in glass vials at −20°C under darkness until analysis. Prior to closing the vials, a stream of N_2_ was passed.

### 2.3 HPLC–PAD analysis

Phenolic compounds in the UAE extract (UAE yarrow), precipitate (yarrow PP), and separator (yarrow Sep) fractions were identified using HPLC−PAD analysis, following the method described by Villalva et al. (2018). For this purpose, an Agilent HPLC 1260 Infinity series system with a PAD system (Agilent Technologies Inc., Santa Clara, CA, United States) was used. The column used was an ACE Excel 3 Super C18 column (150 mm × 4.6 mm, 3 μm particle size) protected by a guard column filled with the same material (10 mm × 3 mm) at 35°C. Samples were dissolved in DMSO, filtered (0.45 μm PVDF filter), and consequently injected (20 µL) for analysis according to the following elution gradient where acidified milli-Q water with formic acid (0.1% v/v) was used as solvent A and acetonitrile (100%) as solvent B: 0–1 min, 100% A; 6 min, 85% A; 21 min, 75% A; 26 min, 65% A; 36–41 min, 50% A; 43–53 min, 0% A; and 55–60 min, 100% A.

Chromatograms were recorded at 280, 320, 340, and 360 nm. Compounds were identified according to a previous HPLC–PAD–MS/MS study carried out by the research group by comparing their accurate mass spectra and their fragmentation pattern with the literature (Villalva et al., 2021). Analytical standards were used, if available, to confirm the correct identification of each compound regarding its retention time and UV−Vis spectrum. Quantification was performed using calibration curves of analytical standards, except for 6-hydroxyluteolin-7-*O*-glucoside and luteolin-*C*-glucoside (luteolin-7-*O*-glucoside standard was used), schaftoside isomer and apigenin-*C*-hexoside-*C*-pentoside (schaftoside), methyl apigenin-glucoside (vitexin), and dihydroxy-trimethoxyflavone, methoxyacacetin, and methoxyflavone (casticin). Ethyl gallate was added as internal standard in each analyzed sample. The results were expressed as mg phenolic compound/g dry sample.

### 2.4 Gas chromatography–mass spectrometry analysis

Essential oils from the total UAE yarrow extract and its fractions, yarrow PP and yarrow Sep, were identified and quantified by gas chromatography (GC)–mass spectrometry (MS)–FID, following the method developed by Villanueva-Bermejo et al. (Villanueva-Bermejo et al., 2017). For this purpose, an Agilent 7890 A system (Agilent technologies, Santa Clara, CA, United States) equipped with a split/splitless injector, FID detector, and mass spectrometer detector (5975C triple-axis) was used. The analyses were performed using an Agilent HP-5MS capillary column (30 m × 0.25 mm i.d., 0.25 µm phase thickness) and helium as inert gas, maintaining a flow rate of 1 mL/min. The chromatographic method started with a temperature of 40°C, which was increased to 150°C at 3°C/min and held at 150°C for 10 min, then increased from 150°C to 300°C with a ramp of 6°C/min, and finally, maintained at 300°C for 1 min. Samples (1 µL) were injected in the spitless mode. The temperatures for the injector, mass spectrometer ion source, interface, and quadrupole were 250°C, 230°C, 280°C, and 150°C, respectively. The mass spectrometer was operated in the electron impact mode with an energy of 70 eV and was used in the total ion current (TIC) mode, and the mass interval scan ranged from 40 to 500 m/z.

Compound identification was carried out by comparing the mass spectral fragmentation patterns with the Wiley 229 mass spectral library, as well as by contrasting their corresponding retention index (RI) with those described in the literature. The results were expressed as the percentage of area contribution of the total amount of identified compounds.

### 2.5 Measurement of the antioxidant activity

Antioxidant activity was determined using ABTS^•+^ assay, according to the protocol (Re et al., 1999). In brief, for the reaction, 10 µL of the samples were added to 990 µL of diluted ABTS^•+^ radical solution. Then, the reaction took place in the dark until the absorbance reached a plateau. After that, the absorbance was measured at 734 nm, and the results were expressed as TEAC values (mmol Trolox/g extract). For this purpose, Trolox was used as the reference standard, with the following regression equation used for TEAC determination: Y = 47.88X + 0.9237 and R^2^ = 0.9943, where X and Y represent mM Trolox and the inhibition percentage, respectively.

### 2.6 Anti-inflammatory activity

The anti-inflammatory activity was measured following the method described by Villalva et al. (2019). In brief, human THP-1 monocytes (ATCC, Manassas, VA, USA) were cultured in RPMI 1640 media with 10% fetal bovine serum (FBS), 100 U/mL penicillin, 100 mg/mL streptomycin, and 2 mM L-glutamine (Gibco, Paisley, UK). Cells were seeded in a 24-well plate at 5 × 10^5^ cells/well and differentiated to macrophages by incubation with 100 ng/mL phorbol 12-myristate 13-acetate (PMA) (Sigma-Aldrich, Madrid, Spain) for 48 h.

The cytotoxic effect of the extracts was tested on differentiated macrophages, following the MTT assay by Mosmann (1983).

For the anti-inflammatory assay, differentiated cells were incubated with 0.05 μg/mL lipopolysaccharide (LPS) from *E. coli* 055:B5 (Sigma-Aldrich, Madrid, Spain) in the presence of the samples for 24 h. Then, the supernatants were collected and kept frozen at −80°C. The release of cytokines TNF-α, IL-1β, and IL-6 was measured in the supernatants of differentiated cells using an ELISA kit (BD Biosciences, Aalst, Belgium), according to the manufacturer’s instructions.

### 2.7 Analysis of the inhibition of CRC cell viability

Colorectal cancer cells DLD1 and SW620 were purchased from the American Type Culture Collection (Manassas, VA, United States) and cultured in DMEM (2 mM glutamine) and 10% fetal bovine serum (LONZA Iberica, S.A) in 5% CO_2_ atmosphere at 37°C and 95% humidity.

DLD1 and SW620 cells were plated in 96-well plates at densities of 8,000 cells/well and 15,000 cells/well, respectively, overnight (o/n) to allow the cells to attach. The next day, the cells were treated for 48 h with UAE yarrow or Sep yarrow extracts dissolved in DMSO diluted in DMEM (Cultek, Madrid, Spain) growth medium at different concentrations. Subsequently, the culture medium was supplemented with 10% sterile filtered MTT at 5 μg/mL. After 3 h, the medium was removed, and the insoluble formazan crystals were dissolved in 100 µL/well of DMSO. Absorbance was measured at 560 nm in a VICTOR Nivo multimode plate reader (PerkinElmer, Madrid, Spain). The inhibition of growth (mitochondrial function) due to extracts was expressed as a percentage of viable cells in experimental wells relative to control wells to calculate IC_50_ values.

### 2.8 RNA isolation and gene expression analysis

CRC cells were plated and, after an overnight incubation, were treated with two different concentrations of each extract based on the previously determined concentrations, inhibiting 50% of cell viability (IC_50_). Non-treated cells were kept as controls. Total RNA was isolated from each condition with the RNeasy Mini Kit (QIAGEN Iberica, Madrid, Spain), following the manufacturer´s instructions. RNA quality and quantity were checked by UV spectroscopy (NanoDrop 2000 Spectrophotometer, Thermo Scientific, Waltham, Massachusetts, United States).

RNA extracted was subsequently used for real-time quantitative polymerase chain reaction (qPCR). In brief, 1 µg of total RNA was reverse-transcribed with the High-Capacity RNA-to-cDNA Master Mix system (Life Technologies, Madrid, Spain). qPCR was performed in the Quant Studio PCR System (Life Technologies) using the Veri Quest SYBR Green qPCR Master Mix, and specific oligos complementary to the genes of interest, including lipid metabolism-related genes—*SREBF1, FASN, ABCA1,* and *SCD1—*and epithelial-to-mesenchymal transition (EMT) genes—*CDH1, ATP1B1, CDH2,* and *Vimentin*—are indicated in Supplementary Table S1. The relative quantification (RQ) of gene expression for each gene was determined following the 2^−ΔΔCt^ Livak method (Livak and Schmittgen, 2001). RQ Manager Software and ExpressionSuite Software (Applied Biosystems, Madrid, Spain) were applied to calculate the relative expression of each gene.

### 2.9 Analysis of mitochondrial respiration by extracellular flux analysis of the oxygen consumption rate

To analyze the effect of the extracts on mitochondrial respiration, we monitored the extracellular flux of the oxygen consumption rate (OCR) (Cell Mito Stress Test), after the injection of several modulators of the electron transport chain, with the XFe96 Cell Bioanalyzer (Seahorse Biosciences-XFe96, Madrid, Spain). Optimal cell density and drug titration were previously determined. Prior to the experiments, the cells were pre-treated with the indicated doses of the extracts for 48 h. Non-treated cells were kept as controls.

In brief, for the Mito Stress assay, CRC cells were seeded onto 96-well XFe96 cell culture microplates at a density of 8,000 cells/well. The next day, the medium was changed to substrate-limited DMEM without glucose, glutamine, and sodium pyruvate (Life Technologies, Madrid, Spain), and then, it was supplemented with 0.5 mM glucose, 1.0 mM GlutaMAX (Life Technologies, Madrid, Spain), 0.5 mM carnitine (Sigma-Aldrich, Madrid, Spain), and 10% FBS fetal bovine serum for 6–8 h. Then, media were changed to XFe DMEM supplemented with 2.5 mM glucose, 0.5 mM carnitine, and 5 mM HEPES and adjusted to pH 7.4.

Cells were incubated for 45 min–1 h at 37°C without CO_2_. Three different modulators of mitochondrial respiration were sequentially injected. After basal OCR determination, oligomycin (1.5 µM), which inhibits ATPase, was injected to determine the amount of oxygen dedicated to ATP production by mitochondria. To determine the maximal respiration rate or spare respiratory capacity, FCCP (carbonyl cyanide-4- (trifluoro-methoxy) phenyl-hydrazone) was injected (0.8 µM) to free the gradient of H^+^ from the mitochondrial intermembrane space and, thus, to activate maximal respiration. Finally, antimycin A and rotenone (0.5 µM) were added to completely inhibit the mitochondrial respiration.

### 2.10 Lipid accumulation: oil red O staining

CRC cells treated for 48 h with the extracts were washed twice with PBS and then fixed with 4% paraformaldehyde (PFA) at room temperature (RT) for 30 min. After washing twice with PBS and with 60% isopropanol, cells were stained with 3 g/L oil red O (in 60% isopropanol) at RT for 30 min. Next, cells were washed thrice with PBS and left to dry at RT for 10 min for visualizing using a microscope (Leica DM 2000 LED, Madrid, Spain). Finally, cells were resuspended in isopropanol, and absorbance was measured at 510 nm in a VICTOR Nivo multimode plate reader (PerkinElmer, Madrid, Spain).

### 2.11 CRC cell adhesion

The effect of the extracts on cell adhesion was analyzed after plating the cells on a Matrigel matrix (0.4 μg/mm^2^) (Sigma-Aldrich, Madrid, Spain). Afterward, 96-well plates (Corning, Somerville, Massachusetts, United States) were coated with 100 µL Matrigel matrix (diluted in 0.1 M NaHCO_3_) and incubated at 4°C O/N. Then, 96-well plates were blocked with 200 µL of sterile adhesion medium (DMEM 0.5% BSA) during 2 h at 37°C, and CRC cells were fluorescently stained with 10 µL of 1 mg/mL BCEBF (Sigma, Madrid, Spain) per 1 mL of DMEM during 30 min at 37°C and 5% CO_2_. Then, cells were harvested with 4 mM EDTA-PBS, and 2.5 × 10^5^ cells were resuspended in 50 µL of the sterile adhesion medium and transferred to each pre-coated well, previously removing the adhesion medium. At this point, cells were treated with the extracts at the indicated concentrations in quadruplicates. Subsequently, cells were incubated during 2 h at 37°C and 5% CO_2_, and after that, non-attached cells were removed by decantation. Wells were washed twice with 100 µL PBS 1X to properly eliminate all non-attached cells, and finally, attached cells were lysed with 50 µL of 10% SDS in PBS. Plates were incubated on a shaker for 20 min at room temperature and in darkness. The fluorescence signal was read with the Spark multimode microplate (TECAN, Chapel Hill, North Carolina, United States) at 436 nm–535 nm excitation–emission, respectively.

### 2.12 Statistical analysis

Statistical analysis of the data was carried out with Statgraphics v. Centurion XVI for Windows software (StatPoint Inc., Warranton, VA, United States). Data were reported as the mean ± standard deviation. A one-way analysis of variance (ANOVA) followed by Fisher’s least significant difference (LSD) test at *p* < 0.05 was used to look for differences between means. All analyses were conducted in triplicate.

RT-qPCR results were analyzed using two-way ANOVA (Bonferroni *post hoc* test). Statistical differences were considered significant when *p*-value was <0.05.

## 3 Results and discussion

### 3.1 SAS fractionation of the UAE yarrow extract and biochemical characterization of the resulting fractions

SAS fractionation of the UAE yarrow extract was carried out at 15 MPa and 40°C. Previous studies performed by the research group indicated that these were the most suitable conditions to obtain a selective fractionation of the different compounds in the extract (Villalva et al., 2019). After the SAS process, two main fractions were obtained: precipitate (yarrow PP) and separator (yarrow Sep) fractions.

As several biological activities of yarrow extracts, including antitumor activity, have been related to phenolic compounds, an extensive characterization of these compounds recovered in the precipitate and separator fractions was carried out by HPLC–PAD. The chromatogram of phenolic compounds of the UAE yarrow extract is shown in Supplementary Figure S1. As depicted in Table 1, most of the phenolic compounds were recovered in the precipitate fraction (yarrow PP), except for the less-polar ones consisting of three methoxylated flavone structures. Particularly, 3,5-DCQA, the most abundant compound in the UAE yarrow extract, showed an enrichment of approximately 2.1 times in the precipitate fraction. Moreover, other abundant phenolics in the extract, such as vicenin II, schaftoside, and luteolin 7-*O-*glucoside, also showed a 1.9-fold enrichment in the precipitate fraction (Yarrow PP). These results are consistent with the lower solubility of the polar phenolic compounds in the SCCO_2_–ethanol mixture, most of them being precipitated and recovered in the precipitate fraction. Giménez-Rota et al. (2019) also reported a lower solubility of the polar phenolic compounds, such as rosmarinic acid, in the SCCO_2_–ethanol mixture (Giménez-Rota et al., 2019).

**TABLE 1:** Phenolic composition of the UAE yarrow extract, yarrow PP (precipitate), and yarrow Sep (separator fraction) (mg compound/g dry sample). Data shown represents the mean ± S.D. (*n* = 3). ^a,b,c^ Different superscript letters denote statistical differences.

Compound	UAE yarrow	Yarrow PP	Yarrow Sep
Caftaric acid	0.10 ± 0.04^b^	0.49 ± 0.05^a^	nd
Chlorogenic acid	2.73 ± 0.26^b^	5.46 ± 0.33^a^	nd
Cryptochlorogenic acid	0.13 ± 0.01^a^	0.12 ± 0.01^a^	nd
Vicenin II	5.42 ± 0.61^b^	10.70 ± 0.64^a^	nd
Caffeic acid	0.18 ± 0.01^a^	0.21 ± 0.05^a^	nd
Schaftoside isomer	2.05 ± 0.24^b^	4.57 ± 0.29^a^	nd
Schaftoside	5.07 ± 0.48^b^	9.76 ± 0.58^a^	nd
Homoorientin	0.32 ± 0.04^b^	0.90 ± 0.06^a^	nd
Apigenin-C-hexoside-C-pentoside	2.56 ± 0.18^b^	5.35 ± 0.31^a^	nd
Luteolin-C-glucoside	2.64 ± 0.22^b^	5.66 ± 0.34^a^	nd
6-Hydroxyluteolin 7-*O*-glucoside	1.85 ± 0.19^b^	3.48 ± 0.22^a^	nd
Rutin	1.24 ± 0.10^b^	3.28 ± 0.20^a^	nd
Vitexin	0.73 ± 0.11^b^	2.10 ± 0.11^a^	nd
Methyl apigenin-hexoside	2.33 ± 0.16^b^	7.46 ± 2.36^a^	nd
Luteolin 7-*O*-glucoside	4.90 ± 0.36^b^	9.65 ± 0.59^a^	nd
Luteolin 7-*O*-glucuronide	0.25 ± 0.08^b^	1.21 ± 0.07^a^	nd
3,4-DCQA	0.86 ± 0.10^b^	1.59 ± 0.08^a^	nd
1,5-DCQA	0.63 ± 0.04^b^	1.20 ± 0.06^a^	nd
3,5-DCQA	9.78 ± 0.76^b^	20.85 ± 1.25^a^	nd
Apigenin 7-*O*-glucoside	0.96 ± 0.20^b^	2.83 ± 0.15^a^	nd
4,5-DCQA	2.39 ± 0.18^b^	5.20 ± 0.81^a^	nd
Luteolin	0.89 ± 0.08^b^	2.04 ± 0.12^a^	nd
Quercetin	0.24 ± 0.03^b^	0.67 ± 0.05^a^	nd
Apigenin	0.25 ± 0.02^b^	0.62 ± 0.05^a^	nd
Diosmetin	0.55 ± 0.10^b^	0.92 ± 0.07^a^	nd
Dihydroxy-trimethoxyflavone	1.64 ± 0.12^b^	1.05 ± 0.05^c^	8.62 ± 0.00^a^
Methoxyacacetin	2.26 ± 0.17^b^	0.36 ± 0.01^c^	17.85 ± 0.00^a^
Methoxyflavone	4.22 ± 0.29^b^	0.21 ± 0.01^c^	34.39 ± 0.09^a^
Σ phenolic compounds	57.17 ± 4.47^b^	107.95 ± 6.78^a^	60.86 ± 0.09^b^

nd: non-detected.

Considering that the main biological activities of yarrow extracts have also been attributed to the components of the essential oil, yarrow PP and yarrow Sep fractions were also analyzed by GC–MS. Table 2 illustrates that essential oil components were mostly recovered in the separator fraction (yarrow Sep); meanwhile, these compounds were not found in the precipitate fraction (yarrow PP). As explained before, this result is in accordance with the higher solubility of essential oil components in the SCCO_2_–ethanol mixture, due to its low polarity. The most abundant identified bioactive compounds were terpenes and terpenoids such as borneol and grandisol, followed by caryophyllene oxide. Although the UAE yarrow extract also presented a similar GM–MS profile, the total chromatographic area (expressed as ∑ AUC) was higher for the separator fraction.

**TABLE 2:** Essential oil compounds identified by GC–MS. RI and peak area contribution (%) of the original UAE extract and the separator fraction.

RI	Compound	UAE yarrow	Yarrow Sep
1,041	Eucalyptol	3.8	1.6
1,049	ɣ-Vinyl-ɣ-valerolactone	1.5	0.7
1,085	Artemisia alcohol	4.5	3.1
1,097	Sabinene hydrate	3.9	4.2
1,103	α-Thujone	3.1	2.7
1,109	Benzeneethanol	1.4	1.0
1,136	Camphor	4.9	3.8
1,158	Borneol	17.5	19.9
1,166	Artenisyl acetate	2.3	1.4
1,167	Terpinen-4-ol	3.5	3.0
1,178	α-Terpineol	6.3	6.2
1,182	trans-Linalool oxide	1.3	1.1
1,196	Grandisol	11.6	14.7
1,204	2-Hydroxycineole	2.0	1.5
1,239	Piperitone	5.9	5.3
1,352	Eugenol	3.0	2.4
1,396	Jasmone	2.5	1.8
1,418	β-Caryophyllene	2.4	2.3
1,577	Caryophyllene oxide	6.8	10.6
1,810	Saussurea lactone	2.9	2.6
1,846	Hexahydrofarnesyl acetone	8.4	10.0
	**∑ AUC**	**1.51E + 06**	**2.04E + 06**

AUC: Area Under the Curve.

Interestingly, yarrow Sep presented a very similar composition to the previously described supercritical extract from yarrow (SFE yarrow) (Mouhid et al., 2019a), including bioactive compounds such as borneol, caryophyllene oxide, eucalyptol, sabinene, and terpineol.

### 3.2 Antioxidant activity of the UAE yarrow extract and its derived SAS fractions

The antioxidant activity of precipitate (yarrow PP) and separator (yarrow Sep) fractions was determined using the ABTS radical method (TEAC value). As it can be observed in Table 3, the precipitate fraction (yarrow PP) showed the highest antioxidant activity; meanwhile, the separator fraction (yarrow Sep) presented the lowest one. These results are in concordance with the enrichment in phenolic compounds found in the precipitate fraction as the antioxidant activity is generally attributed to the presence of these compounds. Thus, Tajner-Czopek et al. (2020) described high antioxidant activity of dicaffeoylquinic acids (Tajner-Czopek et al., 2020) and reported the antioxidant activity of luteolin and its glucosides (Choi et al., 2014).

**TABLE 3:** Antioxidant activity of UAE extract and SAS fractions: yarrow PP (precipitate) and yarrow Sep (separator fractions). Data shown represent the mean ± S.D. (*n* = 3) of three independent experiments.

SAS condition		TEAC (mmol Trolox/g extract)		
Pressure (MPa)	Temperature (°C)	UAE yarrow	Yarrow PP	Yarrow Sep
15	40	0.51 ± 0.03	0.93 ± 0.04	0.15 ± 0.01

### 3.3 Anti-inflammatory activity of the UAE yarrow extract and its SAS fractions

In the first step, the cytotoxicity of the UAE extract and SAS fractions on THP-1/M cells was evaluated using the MTT method. The results indicated that at 30 μg/mL (concentration used to carry out the anti-inflammatory assays), the cell viability was ≥95% for all samples.

**FIGURE 1:**
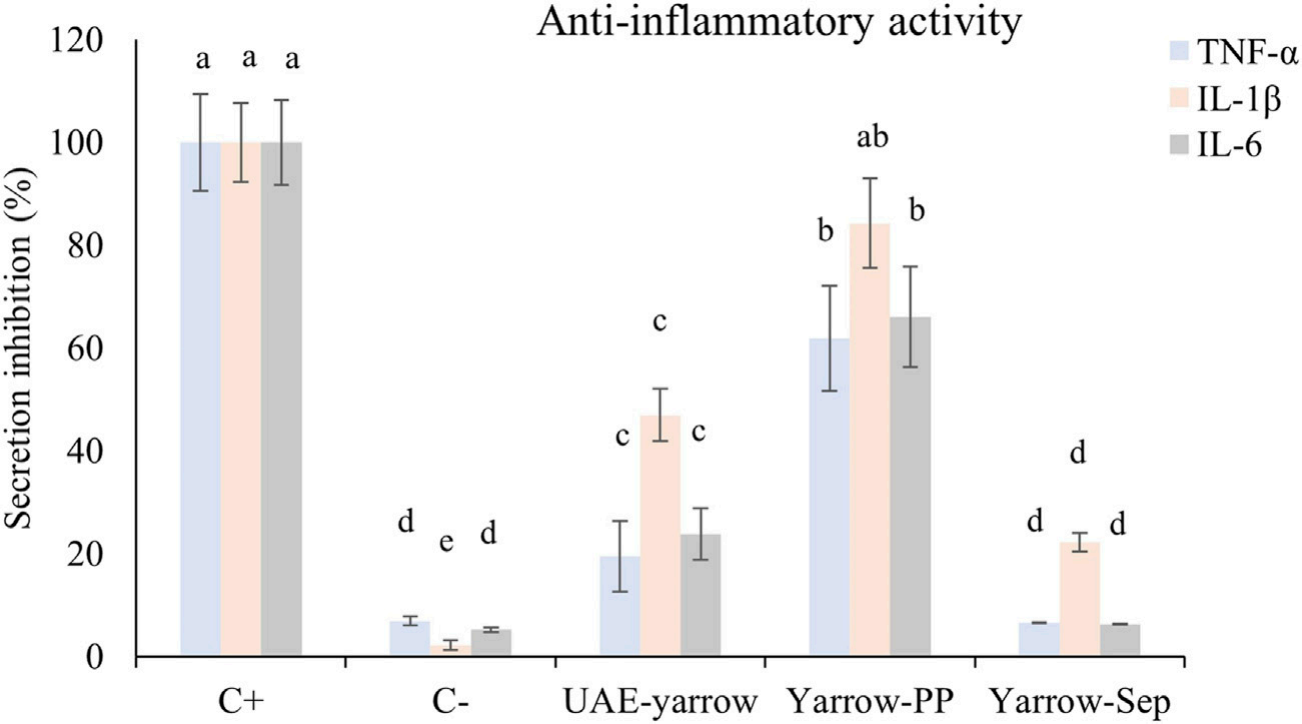
Levels of TNF-α, IL-1β, and IL-6 secreted by THP-1/M activated with LPS in the presence of extract and SAS fractions. ^a–d^ Different letters indicate statistical differences between samples for each interleukin. Significance level at *p* < 0.05 according to Fisher’s least significant difference (LSD) test.

As shown in Figure 1, THP-1/M cells activated with LPS (positive control) presented an increase in the secretion of all pro-inflammatory cytokines studied (TNF-α, IL-1β, and IL-6) compared to non-activated controls (negative control). When the activation of THP-1/M was carried out in the presence of 30 μg/mL of the UAE extract and SAS fractions, a decrease in TNF-α secreted level was observed, compared with levels obtained for positive control. However, while the separator fraction inhibited the secretion up to the levels of the negative control, the precipitate fraction only decreased the secretion by 40%. The UAE yarrow extract showed a TFN-α inhibition value of 80%. Regarding IL-1β, the UAE extract and separator fraction presented a decrease in the secretion of this cytokine, although the greatest decrease was achieved in the separator fraction (80% decreased). The results obtained for the IL-6 release in the presence of samples were similar to those obtained for TNF-α, the separator fraction being the most active in terms of reducing the IL-6 release to basal levels.

Although both SAS fractions presented anti-inflammatory activity, the separator fraction was much more active than the precipitate one and the complete UAE yarrow extract. The anti-inflammatory activity of yarrow PP may be related to its content in phenolic compounds, more specifically with dicaffeoylquinic acids, luteolin, apigenin, and their glycosides. In this regard, Park et al. (2022) indicated that 3,5-DCQA (the main DCQA in the precipitate fraction) treatment reduced LPS-induced pro-inflammatory cytokine expression in microglial cells (Park et al., 2022). Moreover, Caporali et al. (2022) reviewed the anti-inflammatory activity of luteolin and luteolin 7-*O*-glucoside (Caporali et al., 2022). They concluded that these components demonstrated an interesting anti-inflammatory action, both *in vitro* and *in vivo*, mainly in the interaction with JAK/STAT3 and NF-kB.

On the other hand, although yarrow Sep contained a high quantity of methoxylated flavones that have been reported to possess anti-inflammatory activity (Mu et al., 2019), these fractions were mainly enriched in essential oil components, such as borneol, grandisol, and caryophyllene oxide. The anti-inflammatory activity of these terpenes and terpenoids is well known and has been summarized by Kim et al. (2020). Thus, the anti-inflammatory activity found in this fraction may be associated with the presence of several components including phenolic compounds—such as methoxylated flavones—and terpenes and terpenoids—such as borneol, grandisol, and caryophyllene oxide.

### 3.4 Yarrow Sep diminished the cell viability of colorectal cancer cells in a higher manner than UAE yarrow

**FIGURE 2:**
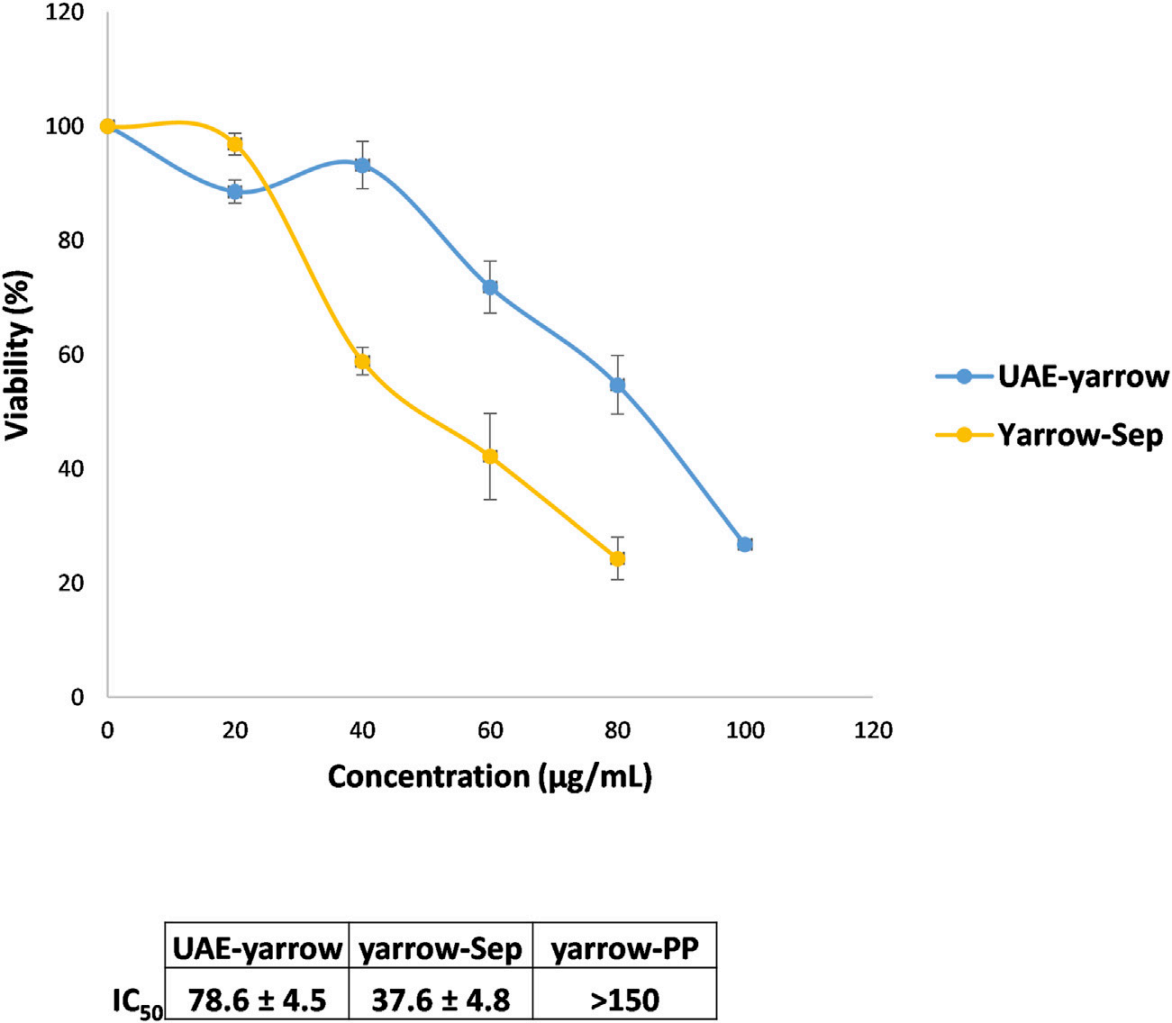
Effect of the yarrow extracts on cell viability (MTT assay) of DLD1 colorectal cancer cells. Dose–response curves of the cell proliferation assay after 48 h of treatment with increasing concentrations of UAE yarrow and yarrow Sep extracts. Data represent the mean ± SEM of three independent experiments, each performed in triplicate.

Next, the effect of the extracts on cell viability of colorectal cancer cells was evaluated. With this purpose, DLD1 colorectal cancer cells were treated for 48 h with the extracts at different concentrations. Meanwhile yarrow Sep compromised DLD1 cell viability higher than UAE yarrow, the IC_50_ values—concentration that inhibits 50% of cell viability—being 37.6 ± 4.8 and 78.6 ± 4.5 for yarrow Sep and UAE yarrow, respectively; yarrow PP did not compromise the cell’s viability at the doses tested (IC_50_ > 150 μg/mL) (Figure 2).

The higher inhibition of the CRC cell viability of yarrow Sep may be related to its higher enrichment in essential oil components, mainly terpenes and terpenoids, compared to the complete UAE yarrow. In this regard, casticin, a methoxylated flavone, and additional essential oil components have been proposed to inhibit the proliferation of several cancer cells, including colorectal cancer cells (Chan et al., 2018; Masyita et al., 2022).

These results are interesting as there is no correlation between the antioxidant and the antiproliferative activities. The antioxidant activity of bioactive compounds is controversial in cancer treatment due to its dual effect, protecting against oxidative stress and DNA damage previous to tumor development but enhancing the redox balance once tumor is established, facilitating cancer cell dissemination. Thus, yarrow Sep inhibited cancer cell proliferation, this activity being independent of its antioxidant activity.

### 3.5 Yarrow Sep inhibited lipid metabolism related targets in CRC cells higher than UAE yarrow

Yarrow Sep, being rich in volatile compounds—such as borneol, caryophyllene oxide, eucalyptol, sabinene, and terpineol, as identified by GC–MS—is also present in a supercritical fluid extract from yarrow (SFE yarrow) with effects on the inhibition of lipid metabolism *in vitro* and in preclinical models of high-fat diet-induced obesity and pancreatic cancer xenografts (Mouhid et al., 2019a; Mouhid et al., 2019b). Next, the effect of yarrow Sep on key lipid metabolism targets implicated in colorectal cancer progression and dissemination was evaluated. A supercritical extract from rosemary (SFE-RE), previously shown to inhibit lipid metabolism in CRC, was used for comparison purposes (Gomez de Cedron et al., 2019b; Bouzas et al., 2022).

**FIGURE 3:**
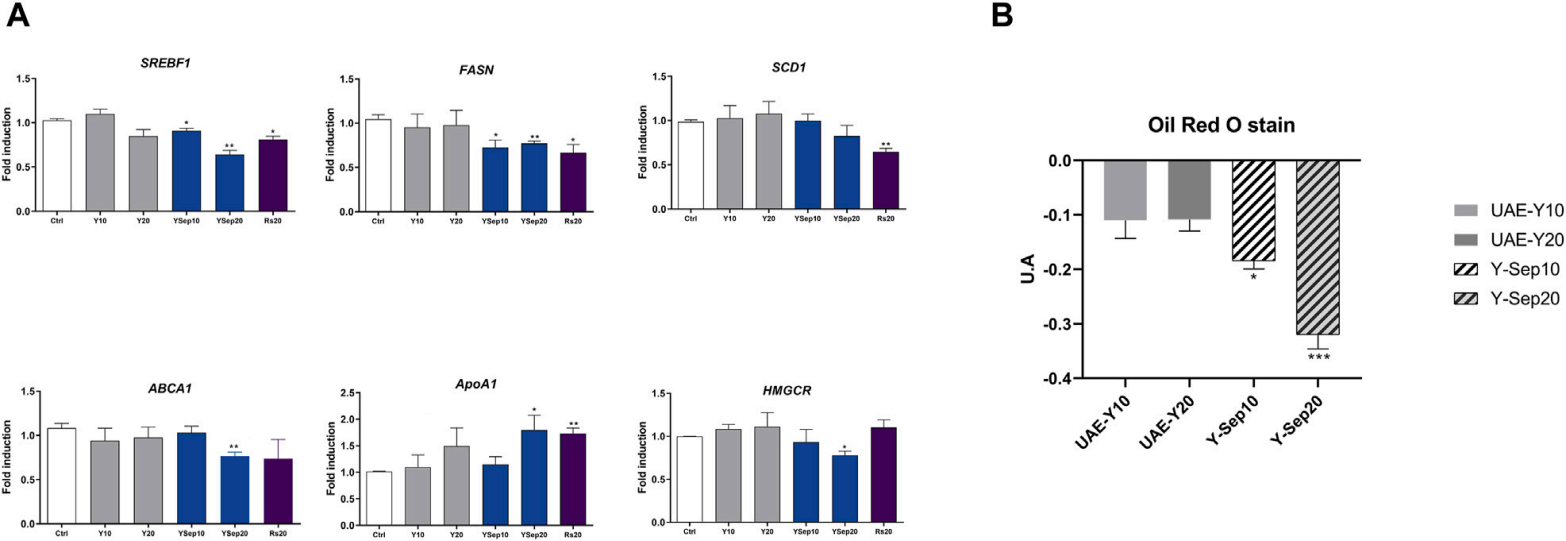
**(A)** Effect of UAE yarrow and yarrow Sep extracts in the expression of lipid metabolic targets in the DLD1 CRC cells. **(B)** Red Oil O staining quantification after treatment with the extracts for 48 h. Asterisks indicate significant statistic differences *p-value <0.05; **p-value <0.01 (three to four replicates, three independent experiments).

As shown in Figure 3A, yarrow Sep significantly inhibited the expression of main drivers of the *de novo* lipogenesis and cholesteroigenesis, *SREBF1*, *FASN*, *ABCA1*, and *HMGCR*. On the contrary, an increase in the expression of *ApoA1*, previously described to counteract the pro-tumoral effect of *ABCA1* in CRC, was found (Aguirre-Portoles et al., 2017). This was also corroborated by the *in vitro* staining of neutral lipids (Figure 3B), where yarrow Sep significantly reduced the neutral lipid content compared to non-treated cells.

These results were also validated in the metastatic CRC cell line SW620, where only yarrow Sep significantly reduced the expression of the master regulator transcription factor *SREBF1* gene expression (*p* < 0.045) (Supplementary Figure S2).

These results suggest that volatile bioactive compounds in yarrow Sep seem to be the main drivers of the inhibition of lipid metabolism targets implicated in the inhibition of CRC cell viability.

### 3.6 Yarrow Sep extract inhibited CRC cell bioenergetics higher than UAE yarrow

As yarrow Sep reduced the intracellular lipid content, next it was investigated if this may be translated into a reduction of cell bioenergetics. Thus, the OCR of CRC cells previously pre-treated with the extracts, UAE yarrow or yarrow Sep, at two different doses, ½ × IC_50_ and 1 × IC_50_, for each extract was evaluated. After 48 h of treatment, the same number of cells were plated in the absence of the extracts to eliminate the effects due to the inhibition of cell viability. Then, cells were exposed to a substrate-limited medium with reduced availability of extracellular fatty acids (FAs) (1% FBS) and glucose (0.5 mM glucose). Then, 0.5 mM carnitine was added to favor the use of intracellular FAs for fatty acid oxidation (FAO).

**FIGURE 4:**
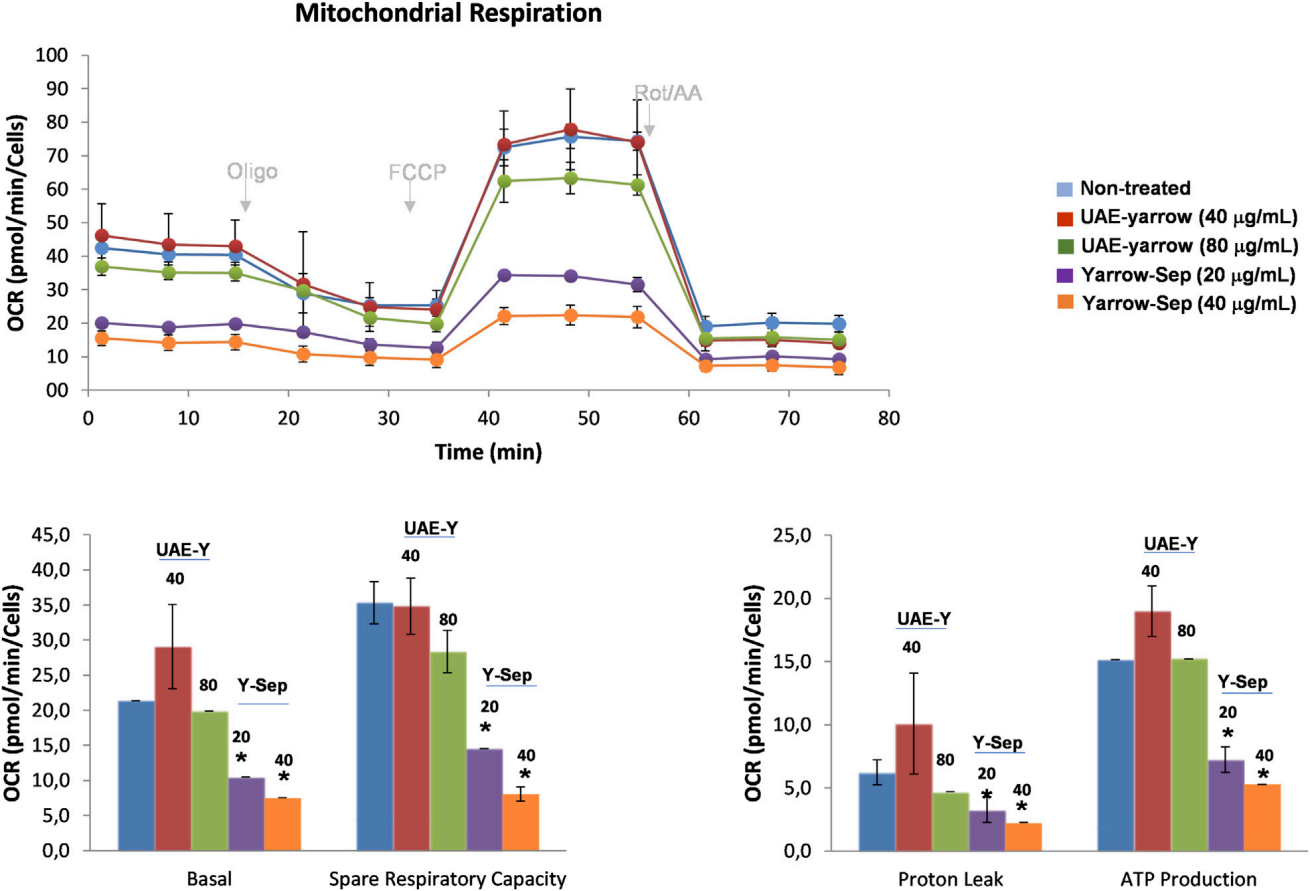
Effect of yarrow UAE and yarrow Sep in the mitochondrial oxidative phosphorylation of CRC cancer cells. Mitochondrial respiration quantification by flux analysis of the oxygen consumption rate (OCR) of DLD1 cells previously pre-treated with yarrow UAE and yarrow Sep extracts for 48 h. The basal respiration rate, spare respiratory capacity, ATP production, and proton leak of 8,000 cells per condition are compared. Data represent the mean ± SEM of four to six replicates. Asterisks indicate statistically significant differences *p < 0.05; **p < 0.01; ***p < 0.005; and ****p < 0.001 relative to the control non-treated cells.

As shown in Figure 4, yarrow Sep inhibited basal respiration rate (BRR) and maximal respiration rates (MRR) and the calculated ATP levels in a dose-dependent manner. Thus, yarrow Sep compromises the oxidative phosphorylation when there is reduced availability of FAs in line with the inhibition of lipid metabolism targets by the extract. These results were also validated in the metastatic CRC cell line SW620, where only yarrow Sep significantly reduced the respiration rates, both in basal and stressed conditions (Supplementary Figure S3).

As far as our knowledge, no previous studies have evaluated the effects of yarrow-derived extracts on the inhibition of CRC cell bioenergetics.

### 3.7 Yarrow Sep extract inhibited the EMT process in a higher manner than UAE yarrow

Finally, due to metastasis being the last step of cancer and the described role of lipid metabolism in the promotion of cancer dissemination (Chaffer and Weinberg, 2011), the effect of UAE yarrow and yarrow Sep on the key targets of EMT was explored.

**FIGURE 5:**
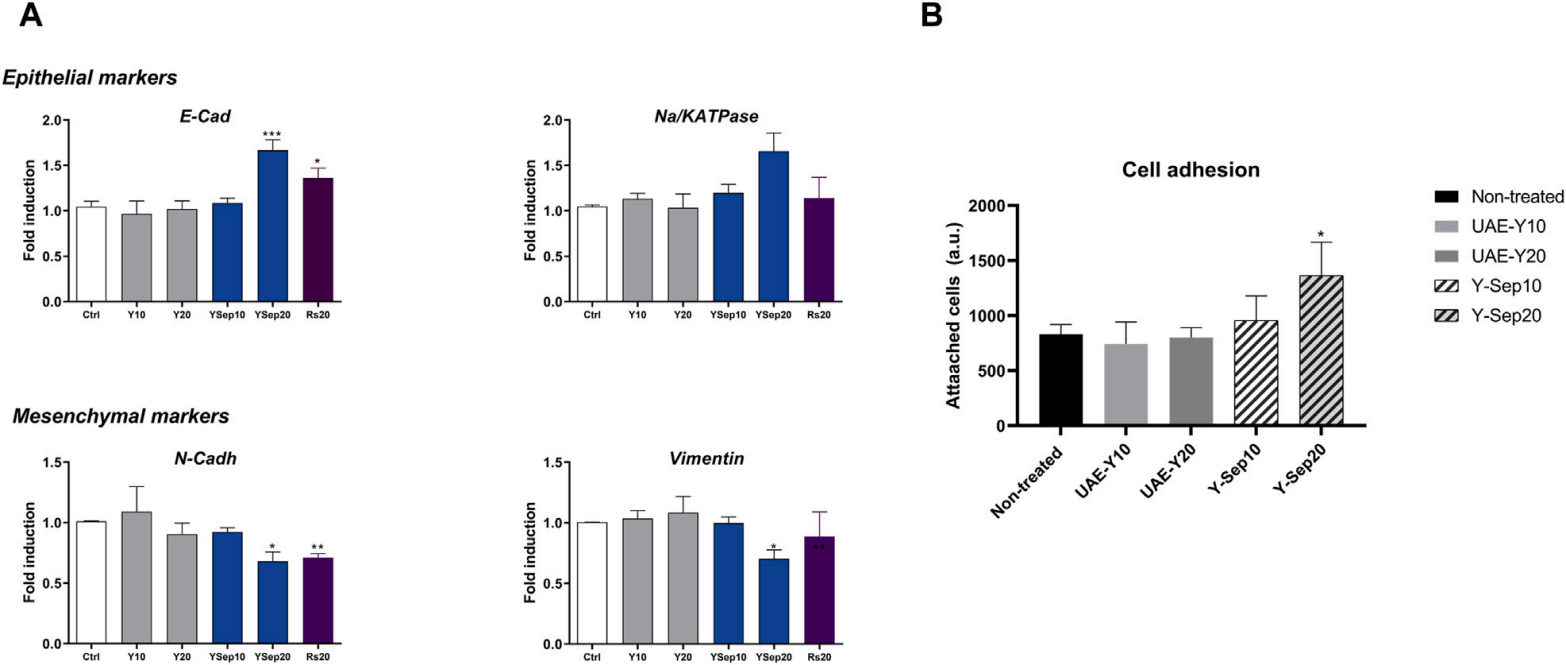
**(A)** Effect of yarrow UAE and yarrow Sep extracts in the expression of EMT targets in the DLD1 CRC cells. **(B)** *In vitro* functional cell-based assay for the cell adhesion capacity of colorectal cancer cells. Asterisks indicate statistically significant differences *p < 0.05; **p < 0.01; ***p < 0.005; and ****p < 0.001 relative to the control non-treated cells (three replicates, three independent experiments).

As shown in Figure 5A, yarrow Sep significantly inhibited the expression of the mesenchymal markers, vimentin and N-cadherin, and upregulated the expression of the epithelial marker E-cadherin compared to non-treated cells. The *in vitro* analysis of cell adhesion functional assay also showed the effect of yarrow Sep significantly augmenting the cell adhesion capacity of CRC cells compared to control non-treated cells (Figure 5B).

These results were also observed in the metastatic CRC cell line SW620, where only yarrow Sep significantly augmented the expression of E-cadherin, although no effects were found on the inhibition of mesenchymal markers. (Supplementary Figure S4).

These results suggest that yarrow Sep inhibits the EMT process at least partially mediated by the effects on the inhibition of key lipid metabolism targets implicated in cancer dissemination.

## 4 Highlights and conclusion

SAS precipitation was successfully applied to fractionate UAE yarrow into two extracts with different polarities: the yarrow precipitate (yarrow PP), rich in polar phenolic bioactive compounds, and yarrow separator (yarrow Sep), rich in essential oil components.

Most of the UAE yarrow phenolic compounds were recovered in the precipitate fraction yarrow PP, highlighting 3,5-DCQA. The yarrow Sep fraction was enriched in essential oil components, such as borneol, grandisol, and caryophyllene oxide.

Yarrow PP exerted the highest antioxidant activity, even higher than the complete UAE yarrow; meanwhile, yarrow Sep showed the highest anti-inflammatory activity, even higher than the complete UAE yarrow.

Yarrow Sep inhibited key lipid metabolic targets shown to be implicated in CRC dissemination and poorer prognosis. More specifically, yarrow Sep significantly inhibited the expression of the main drivers of the *de novo* lipogenesis and cholesterogenesis—*SREBF1*, *FASN*, *ABCA1*, and *HMGCR*—in line with the reduction of the neutral lipid content quantified in yarrow Sep-treated cells compared to non-treated cells.

These results suggest that volatile bioactive compounds present in the yarrow Sep fraction may be responsible for the inhibition of lipid metabolism in CRC.

Finally, yarrow Sep targeted epithelial-to-mesenchymal transition markers—*CDH1*, *ATP1B1*, *CDH2*, and *Vimentin*—augmenting CRC cell adhesion.

In summary, SAS technology was applied to provide a novel combination of bioactive compounds, yarrow Sep, which merits further investigation to be proposed as a potential complementary nutraceutical in the treatment of CRC.

## Data Availability

The original contributions presented in the study are included in the article/Supplementary Material; further inquiries can be directed to the corresponding authors.

## Supplementary material

Supplementary figure S1 Chromatogram of the phenolic compounds of the UAE yarrow extract (λ = 280 nm).

Supplementary figure S2 Effect of UAE yarrow and yarrow Sep extracts in the inhibition of the gene expression of the master regulator of *de novo* lipogenesis *SREBF1* in the metastasis CRC cell line SW620. Asterisks indicate statistically significant differences **p* < 0.05; ***p* < 0.01; ****p* < 0.005; and *****p* < 0.001 relative to the control non-treated cells (three replicates, three independent experiments).

Supplementary figure S3 Effect of yarrow UAE and yarrow Sep in the mitochondrial oxidative phosphorylation of SW620 metastatic CRC cells. The basal respiration rate, spare respiratory capacity, ATP production, and proton leak of 8,000 cells per condition are compared. Data represent the mean ± SEM of four to six replicates.

Supplementary figure S4 Effect of UAE yarrow and yarrow Sep extracts in the expression of the epithelial markers E-cadherin in the metastatic CRC cell line SW620. Asterisks indicate statistically significant differences **p* < 0.05; ***p* < 0.01; ****p* < 0.005; and *****p* < 0.001 relative to the control non-treated cells (three replicates, three independent experiments).

Supplementary table S1 List of oligos (forward and reverse) to assay lipid metabolism gene expression and epithelial to mesenchymal transition genes.
