# Supplementary figures and images for: Novel bioactive extract from yarrow obtained by the supercritical antisolvent-assisted technique inhibits lipid metabolism in colorectal cancer
Source: Front Bioeng Biotechnol. 2024 Mar 21;12:1256190. doi: 10.3389/fbioe.2024.1256190 (PMC10991822; doi:10.3389/fbioe.2024.1256190)

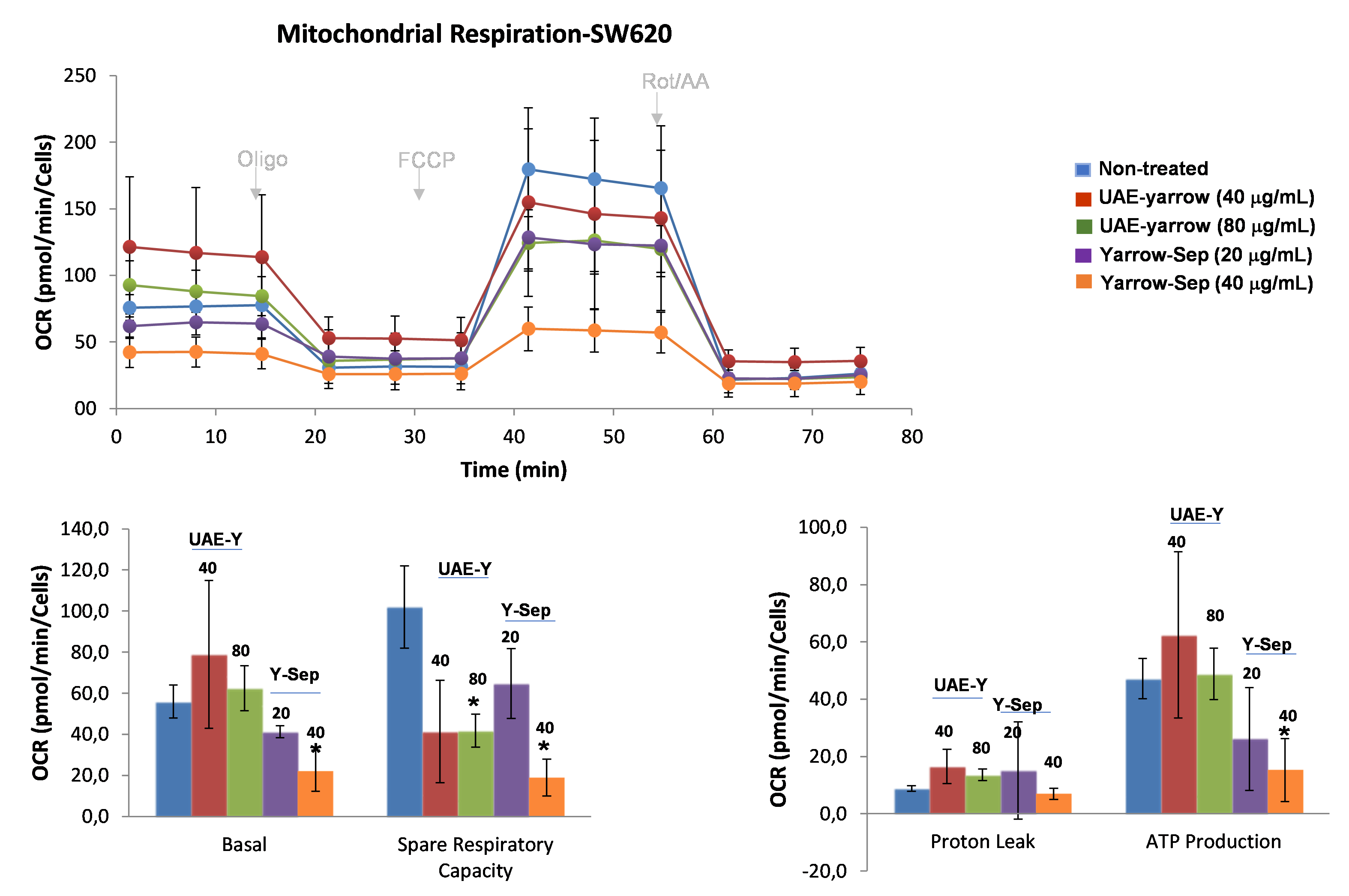

Supplement: Supplementary file 1 [file Image3.TIF]

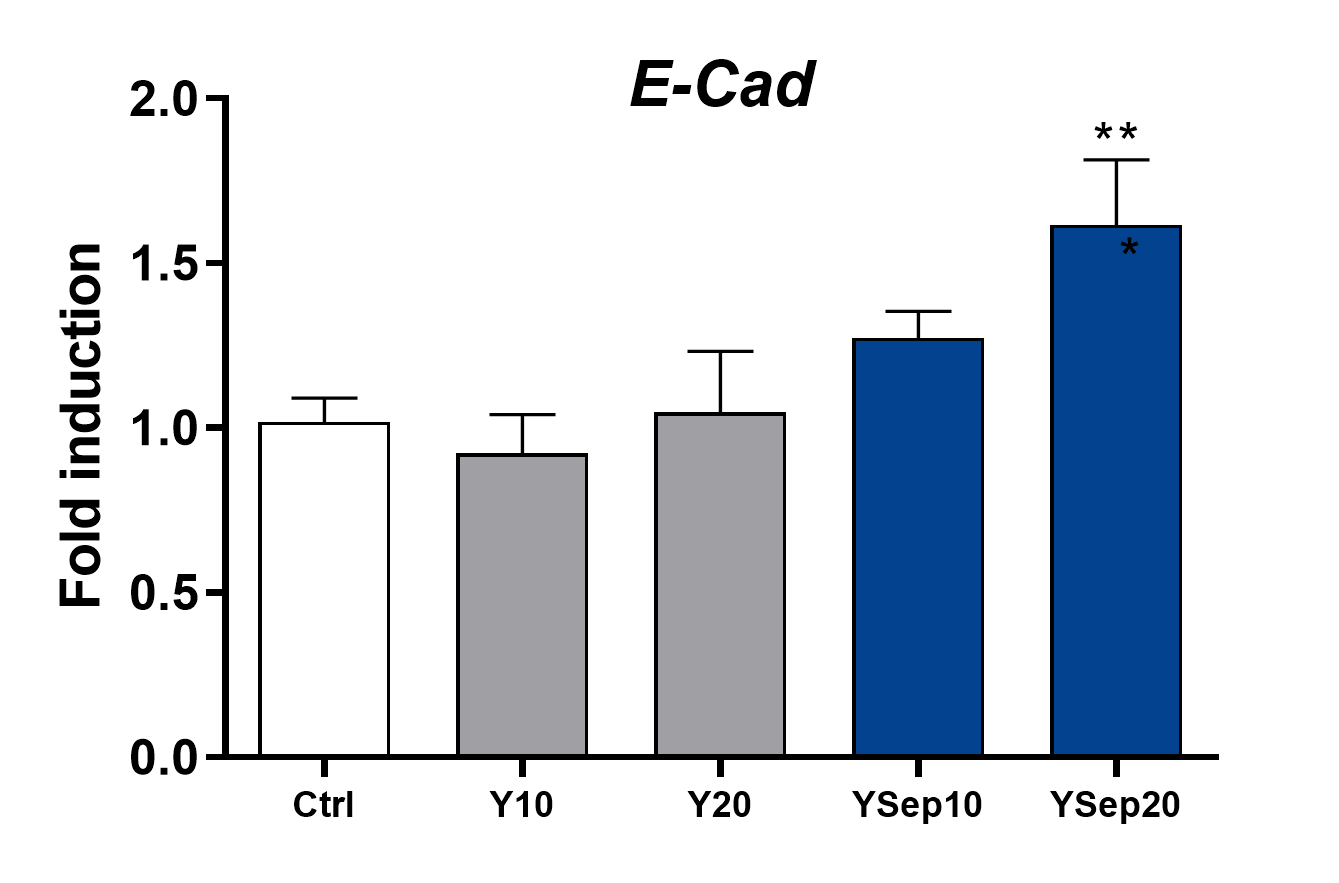

Supplement: Supplementary file 2 [file Image4.TIF]

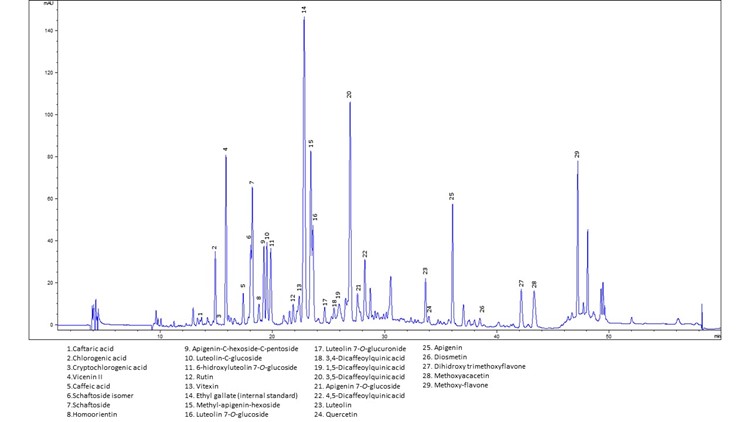

Supplement: Supplementary file 3 [file Image1.JPEG]

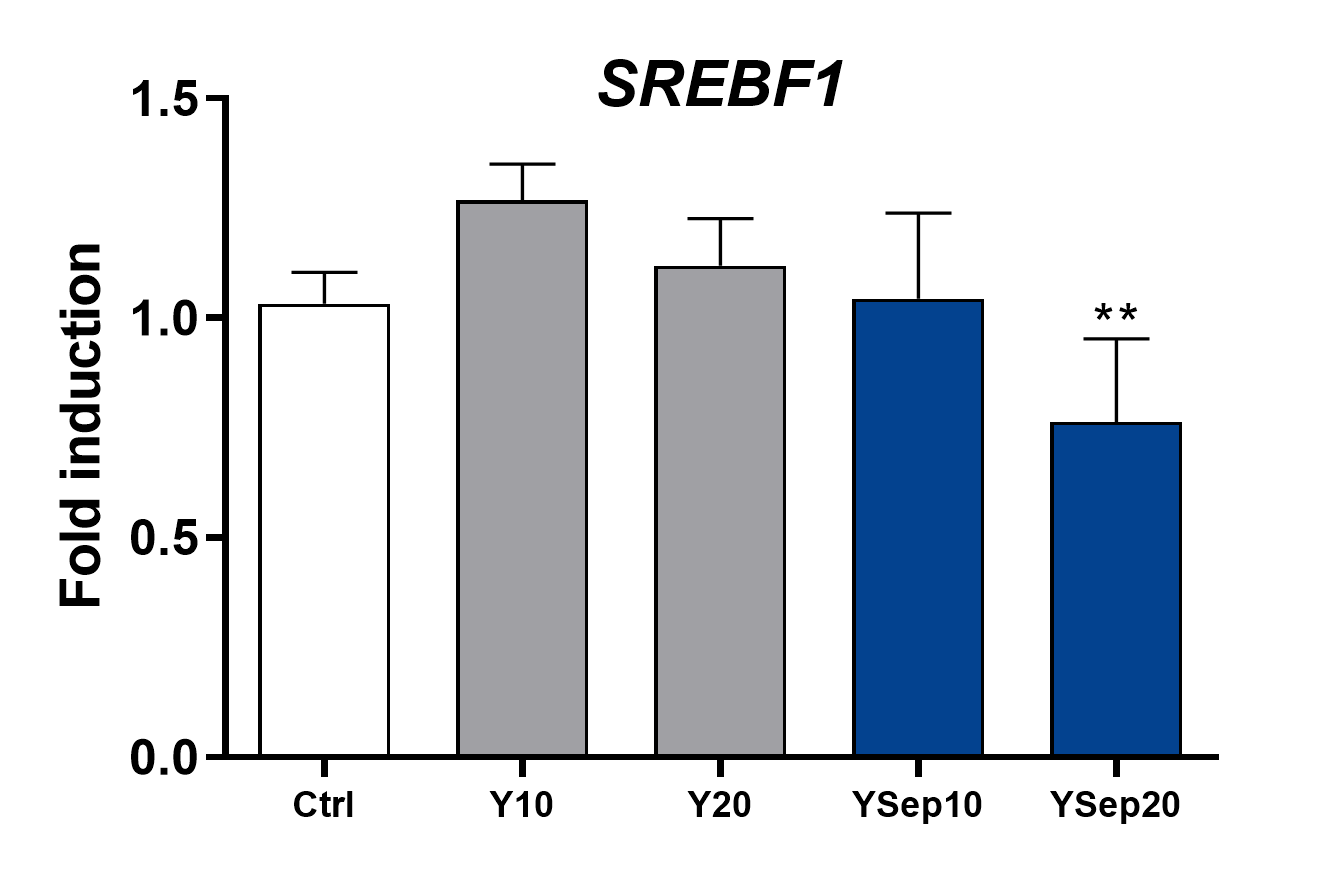

Supplement: Supplementary file 4 [file Image2.TIF]
